# Identification of Critical m^6^A RNA Methylation Regulators with Prognostic Value in Lower-Grade Glioma

**DOI:** 10.1155/2021/9959212

**Published:** 2021-06-08

**Authors:** Jianglin Zheng, Xuan Wang, Yue Qiu, Minjie Wang, Hao Yu, Zijie Zhou, Zhipeng Wu, Xiaobing Jiang

**Affiliations:** ^1^Department of Neurosurgery, Union Hospital, Tongji Medical College, Huazhong University of Science and Technology, Wuhan, Hubei, China; ^2^Department of Otolaryngology, Union Hospital, Tongji Medical College, Huazhong University of Science and Technology, Wuhan, Hubei, China

## Abstract

Increasing evidences have revealed that N^6^-methyladenosine (m^6^A) RNA methylation regulators participate in the tumorigenesis and development of multiple tumors. So far, there has been little comprehension about the effects of m^6^A RNA methylation regulators on lower-grade gliomas (LGG). Here, we systematically investigated the expression profiles and prognostic significance of 36 m^6^A RNA methylation regulators in LGG patients from the TCGA and CGGA databases. Most of the m^6^A RNA methylation regulators are differentially expressed in LGG tissues as compared with normal brain tissues and glioblastoma (GBM) tissues. The consensus clustering for these m^6^A RNA methylation regulators identified three clusters. Patients in cluster 3 exhibited worse prognosis. In addition, we constructed an m^6^A-related prognostic signature, which exhibited excellent performance in prognostic stratification of LGG patients according to the results of the Kaplan-Meier curves, ROC curves, and univariate and multivariate Cox regression analyses. In addition, a significant correlation was observed between the m^6^A-related prognostic signature and the immune landscape of the LGG microenvironment. The high-risk group exhibited higher immune scores, stromal scores, and ESTIMATE scores but lower tumor purity and lower abundance of activated NK cells. Moreover, the expression level of immune checkpoints was positively correlated with the risk score. To conclude, the current research systematically demonstrated the prognostic roles of m^6^A RNA methylation regulators in LGG.

## 1. Introduction

Gliomas constitute the frequent intracranial malignant tumors, showing significant heterogeneity with respect to tumor biological behavior for different grade gliomas [1, 2]. Despite the less malignancy of low-grade gliomas (LGG) compared with glioblastomas (GBM), tumor recurrence and malignant progression seem to be ineluctable for LGG patients even with the standard treatments, including surgical resection, radiotherapy, and chemotherapy [3]. So far, we have made a considerable advance in understanding the genetic landscape of LGG, and favorable treatment options remain insufficient. Hence, exploring new prognostic biomarkers or treatment targets is of great clinical significance for LGG patients.

RNA modification is an emerging field of epigenetics. Among more than 150 RNA modifications, N^6^-methyladenosine (m^6^A) constitutes the most frequent type in eukaryotes [4, 5] and is critically important for gene expression regulation [6, 7]. As a reversible and dynamic process, m^6^A RNA methylation is regulated by m^6^A RNA methylation regulators, including methyltransferases, demethylases, and binding proteins, namely, “writers”, “erasers”, and “readers”, respectively. Increasing evidences have suggested that m^6^A modification contributes to the malignant biological behavior of multiple cancers [8, 9]. The prognostic value of m^6^A RNA methylation regulators has also been confirmed in head and neck squamous cell carcinoma [10], thyroid carcinoma [11], breast cancer [12], hepatocellular carcinoma [13], gastric cancer [14], etc. However, no literatures have comprehensively evaluated the prognostic role of m^6^A RNA methylation regulators in LGG.

Here, the expression profile and prognostic significance of 36 m^6^A RNA methylation regulators were systematically evaluated in LGG using the data from The Cancer Genome Atlas (TCGA) dataset and Chinese Glioma Genome Atlas (CGGA) dataset. In addition, we constructed an m^6^A-related prognostic signature with robust ability in predicting survival outcomes of LGG patients.

## 2. Materials and Methods

### 2.1. Data Acquisition

Total 1013 LGG patients with RNA-seq transcriptome data and corresponding clinicopathological features were identified in public databases, including 423 cases from the TCGA dataset and 590 cases from the CGGA dataset. Patients without survival information or OS <30 days, or without definitive histopathological diagnosis had been excluded from further evaluation. These two datasets were used as the training cohort (TCGA) and validation cohort (CGGA). The RNA-seq transcriptome data of 160 GBM tissues and 1152 normal brain tissues were obtained from the TCGA dataset and the Genotype-Tissue Expression (GTEx) database, respectively. All RNA-seq transcriptome data were harmonized using quantile normalization and SVAseq-based batch effect removal [15]. The clinicopathological features of included LGG patients were summarized in Table 1.

### 2.2. Screening of m^6^A RNA Methylation Regulators with Differential Expression Profiles

According to published articles and reviews [16, 17], thirty-six m^6^A RNA methylation regulators with obtainable expression data were identified in the TCGA, CGGA, and GTEx datasets (Table 2). The expression of these 36 m^6^A RNA methylation regulators was compared in LGG tissues with normal brain tissues and glioblastoma tissues and with different WHO grades.

### 2.3. Consensus Clustering

Interactions among m^6^A RNA methylation regulators were analyzed utilizing the STRING database. Using the R package “ConsensusClusterPlus” [18], distinct clusters of LGG patients were identified based on the expression levels of 36 m^6^A RNA methylation regulators. Principal Component Analysis (PCA) was performed to confirm the reliability of clustering results [19]. The differentially expressed genes (DEGs) in different clusters were determined (∣log2FC | >2 and adjusted *p* < 0.05) and functionally annotated by the Gene Ontology (GO) analysis and the Kyoto Encyclopedia of genes and Genomes (KEGG) pathway analysis.

### 2.4. Construction and Validation of the m^6^A-Related Prognostic Signature

Univariate Cox regression was performed to identify the overall survival- (OS-) associated m^6^A RNA methylation regulators (*p* < 0.05), which were subsequently incorporated into the least absolute shrinkage and selection operator (LASSO) Cox regression. A total of nine m^6^A RNA methylation regulators with LASSO coefficients were identified. The calculation formula of risk score is shown below:

(1) $$\begin{array}{c} \text{Risk score}=\overset{n}{\underset{i=1}{\sum }}{\text{Coef}}_{i}*x_{i.} \end{array}$$

where *x*_*i*_ and Coef_*i*_ refer to the expression level of selected m^6^A RNA methylation regulator and corresponding LASSO coefficient, respectively. The median risk score was used for the high-/low-risk grouping of LGG patients. The Kaplan-Meier curve with log-rank test was generated by using the R package “survminer” for the comparison of OS between the high- and low-risk groups. The ROC curve analysis was utilized to evaluate the prediction accuracy of the m^6^A-related prognostic signature via the R package “timeROC”. All these tests were performed simultaneously in the training and validation cohorts.

### 2.5. Establishment and Evaluation of a Nomogram

By employing the R package “rms”, “regplot”, and “Hmisc”, a nomogram was established based on the independent prognostic indicators in the training cohort, which were determined through univariate and multivariate Cox regression analyses. The availability of this nomogram was evaluated by the C-indices [20] and calibration curves.

### 2.6. Evaluation of the Immune Landscape

The immune scores, stromal scores, ESTIMATE scores, and tumor purity of each LGG patients were calculated using the ESTIMATE algorithm via the R package “estimate” [21]. The abundance of 22 immune cells was calculated through the CIBERSORT algorithm with 1,000 permutations [22]. Patients with CIBERSORT *p* ≥ 0.05 were excluded from the subsequent analysis.

### 2.7. Statistical Analysis

The preprocessing of RNA-seq transcriptome data was performed using the PERL programming language (version 5.32.0). The R software (version 4.0.2) was applied for all statistical analyses and graph visualization. The student *t*-test or one-way ANOVA test was utilized to compare the continuous variables with normal distribution between two groups or more than two groups. The Wilcoxon test was performed to determine the differences between subgroups in the expression levels of m^6^A RNA methylation regulators and the abundance of 22 immune cells. The “Spearman” method was used to calculate the correlations between the expression of m^6^A RNA methylation regulators and between the expression of immune checkpoints and risk scores. Two-tailed *p* < 0.05 was considered statistically significant.

## 3. Results

### 3.1. The Expression Profiles of m^6^A RNA Methylation Regulators in LGG

**Figure 1:**
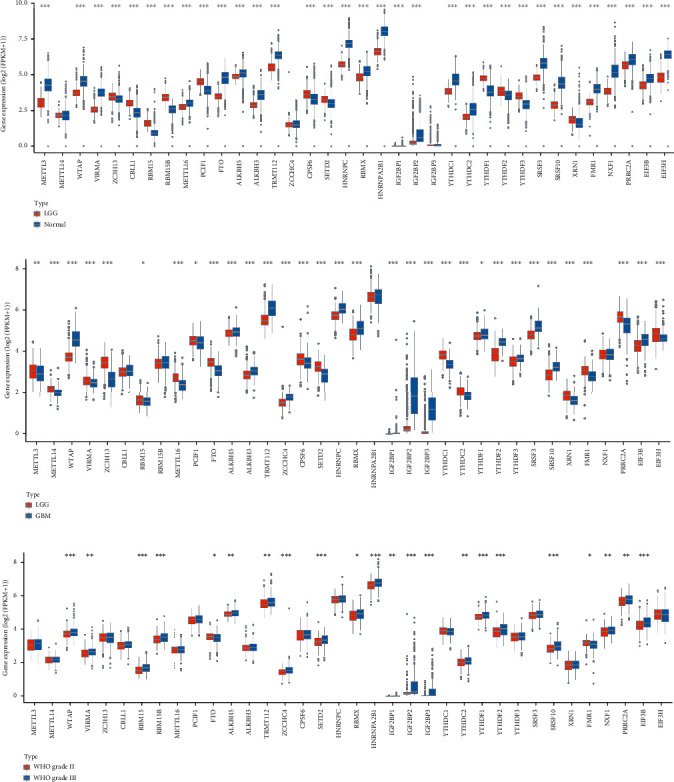
The expression profiles of m^6^A RNA methylation regulators across tissues. (a) The expression comparison of m^6^A RNA methylation regulators between LGG and normal tissues. (b) The expression comparison of m^6^A RNA methylation regulators between LGG and GBM tissues. (c) The expression comparison of m^6^A RNA methylation regulators between WHO grade II and III. ^∗^*p* < 0.05, ^∗∗^*p* < 0.01, ^∗∗∗^*p* < 0.001.

Among 36 m^6^A RNA methylation regulators, twenty-two upregulated genes and eleven downregulated genes were identified in LGG tissues compared with normal brain tissues (Figure 1(a)). Compared with GBM tissues, the expression levels of 16 m^6^A RNA methylation regulators were upregulated, while the expression levels of 15 m^6^A RNA methylation regulators were downregulated in LGG tissues (Figure 1(b)). In addition, there were also large variations in the expression profiles of m^6^A RNA methylation regulators between different histological grades. A total of 22 m^6^A RNA methylation regulators were differentially expressed between WHO grade II and III (Figure 1(c)). Altogether, these results suggested that the m^6^A RNA methylation regulators may play a vital and particular role in the tumorigenesis and progression of LGG.

**Figure 2:**
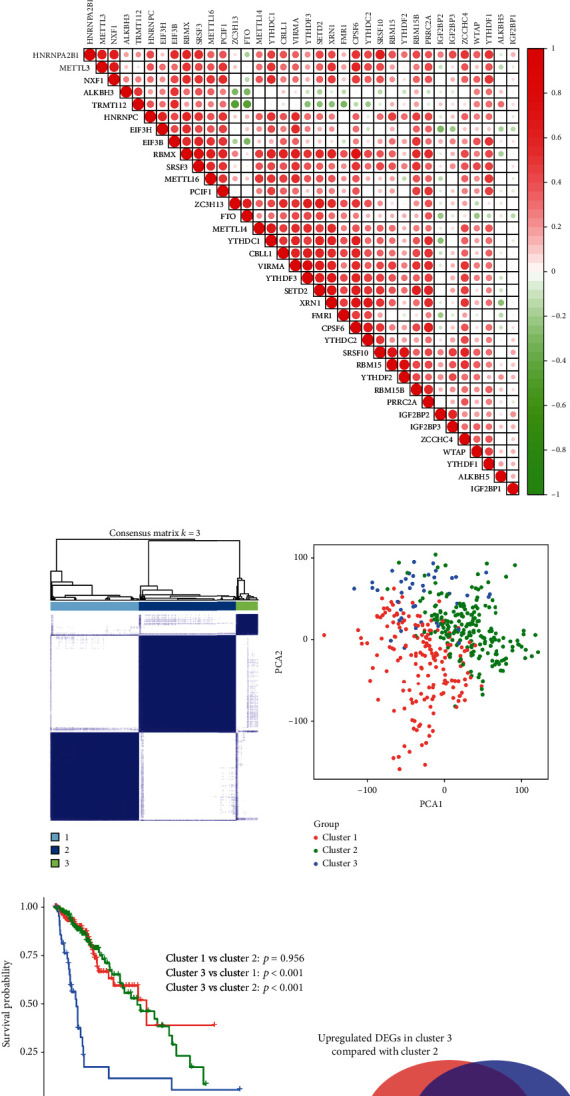
Interaction among m^6^A RNA methylation regulators and consensus clustering of LGG patients in the TCGA cohort. (a) Protein-protein interaction (PPI) network of m^6^A RNA methylation regulators. Elements not connected to others are hidden. (b) Correlation of m^6^A RNA methylation regulators. (c) Consensus clustering matrix for optimal *k* = 3. (d) PCA of the RNA expression profile. (e) The Kaplan-Meier curve for LGG patients in cluster 1/2/3. (f) 303 upregulated overlapping DEGs between clusters 3/1 and 3/2. (g) Gene Ontology biological processes of upregulated overlapping DEGs. (h) KEGG pathway analysis of upregulated overlapping DEGs.

The interactions among 36 m^6^A RNA methylation regulators were presented in the protein-protein interaction (PPI) network and coexpression analyses. Noticeably, “writers” had a wide range of interactions with other m^6^A RNA methylation regulators, while “erasers” were less connected with others (Figure 2(a)). Further coexpression analyses showed most m^6^A RNA methylation regulators were positively correlated with other regulators, and negative coexpression relationships were underrepresented (Figure 2(b)).

### 3.2. Three Clusters of LGG Patients with Distinct Prognosis

According to the similarity in 36 m^6^A RNA methylation regulators expression and the proportion of ambiguous clustering measure, *k* = 3 was picked as the most appropriate value in the TCGA cohort (Figure 2(c) and Supplementary Figure S1). As a result, 423 LGG patients were categorized into three clusters, called cluster 1 (*n* = 180), cluster 2 (*n* = 200), and cluster 3 (*n* = 43). PCA for total transcriptomic data revealed prominent differences in distribution among three clusters, which confirmed the validity of consensus clustering (Figure 2(d)). Then, the survival analysis showed patients in cluster 3 had obviously shorter OS than those in cluster 1 and cluster 2 (Figure 2(e)). To identify the different biological processes in cluster 3 compared with cluster 1 and cluster 2, a total of 303 upregulated overlapping DEGs were identified in cluster 3 (Figure 2(f)) and were functionally annotated by the GO and KEGG pathway analyses. The results indicated that upregulated DEGs were enriched in an extracellular matrix organization, regulation of vasculature development, regulation of angiogenesis, and nuclear division, which were malignancy-related biological processes (Figure 2(g)). Then, the KEGG pathway analysis similarly exhibited the significant enrichment of malignancy-related pathways, including PI3K-Akt pathway, focal adhesion, proteoglycans in cancer, and ECM-receptor interaction (Figure 2(h)).

### 3.3. Construction and Validation of the m^6^A-Related Prognostic Signature

**Figure 3:**
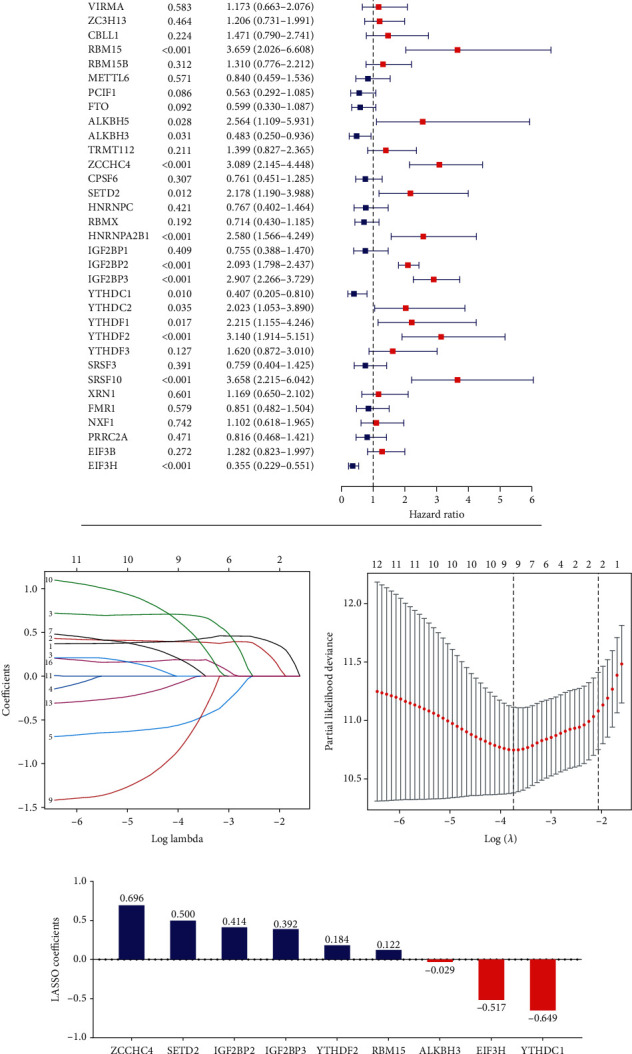
Prognostic value of m^6^A RNA methylation regulators and construction of the m^6^A-related prognostic signature. (a) Overall survival- (OS-) related m^6^A RNA methylation regulators in TCGA cohort. (b, c) LASSO analysis with minimal lambda value. (d) LASSO coefficients of nine m^6^A RNA methylation regulators.

Based on the results of Univariate Cox regression, fourteen m^6^A RNA methylation regulators (RBM15, ALKBH5, ALKBH3, ZCCHC4, SETD2, HNRNPA2B1, IGF2BP2, IGF2BP3, YTHDC1, YTHDC3, YTHDF1, YTHDF2, SRSF10, and EIF3H) were significantly associated with the OS of LGG patients (Figure 3(a)). Subsequently, the LASSO Cox regression for those 14 m^6^A RNA methylation regulators was carried out (Figures 3(b) and 3(c)). A total of 9 m^6^A RNA methylation regulators (ZCCHC4, SETD2, IGF2BP2, IGF2BP3, YTHDF2, RBM15, ALKBH3, EIF3H, and YTHDC1) stood out as the bases of constructing the m^6^A-related prognostic signature (Figure 3(d)). The risk score for each LGG patient was calculated by summing the product of the expression level of each selected m^6^A RNA methylation regulator and corresponding LAASO coefficient. The median risk score was applied to stratify LGG patients into high-/low-risk groups.

**Figure 4:**
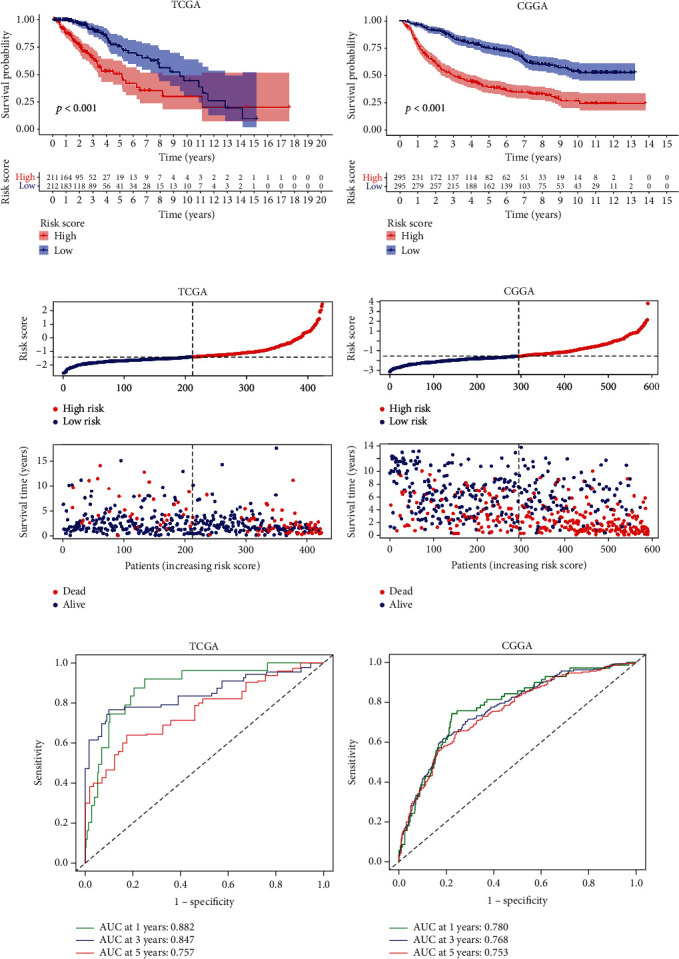
Validation of the m^6^A-related prognostic signature. (a, b) The Kaplan-Meier curves for survival in the TCGA and CGGA cohorts. (c, d) The distribution plots of the risk score and survival status in the TCGA and CGGA cohorts. (e, f) The receiver operating characteristic (ROC) curve analyses of the prognostic FRLS in predicting 1-, 3-, and 5-year overall survival (OS) in the TCGA and CGGA cohorts.

The prognostic value of the m^6^A-related prognostic signature for LGG patients was evaluated in the TCGA cohort and the CGGA cohort, respectively. The Kaplan-Meier curves showed that patients in the high-risk group exhibited shorter OS than those in the low-risk group (Figures 4(a) and 4(b)). The distribution plots of the risk score and survival status revealed that the higher the risk score, the more deaths of LGG patients (Figures 4(c) and 4(d)). Moreover, the high accuracy of this m^6^A-related prognostic signature in predicting 1-, 3-, and 5-year OS was confirmed by the area under the receiver operating characteristic (ROC) curve (AUC). The AUCs of 1-, 3-, and 5-year OS in the TCGA cohort were 0.882, 0.847, and 0.757, respectively (Figure 4(e)), and in the CGGA cohort were 0.780, 0.768, and 0.753, respectively (Figure 4(f)). Overall, the above results all agreed that the m^6^A-related prognostic signature could accurately and stably predict the survival outcome of LGG patients.

### 3.4. Associations between the Risk Score and Clinicopathological Features

**Figure 5:**
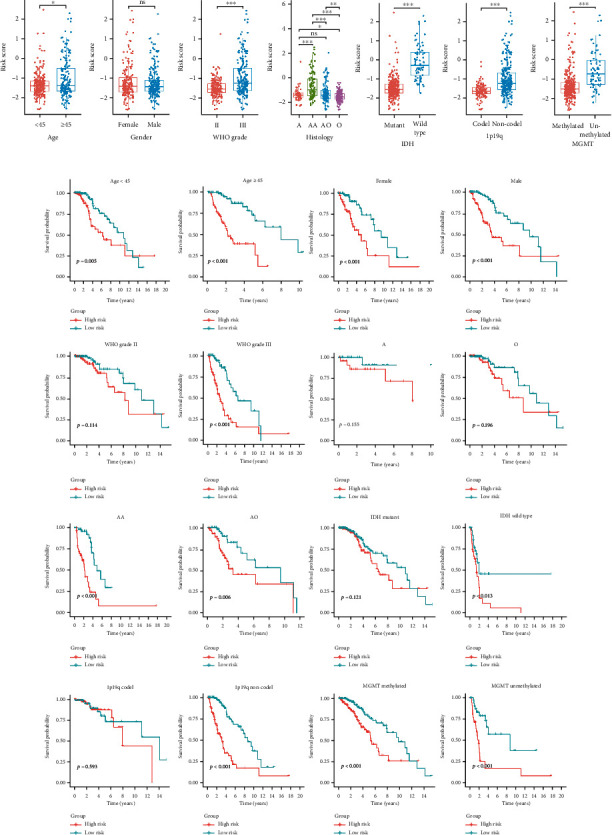
Correlation analysis between the m^6^A-related prognostic signature and clinicopathological features in the TCGA cohort. (a) Different levels of risk scores in LGG patients stratified by age, gender, WHO grade, histology, IDH status, 1p19q codeletion, and MGMT methylation status. (b) The Kaplan-Meier curves for subgroup survival analysis. A: astrocytoma; O: oligodendroglioma; AA: anaplastic astrocytoma; AO: anaplastic oligodendroglioma/oligoastrocytoma. ^∗^*p* < 0.05, ^∗∗^*p* < 0.01, ^∗∗∗^*p* < 0.001; ns: no significance.

The levels of risk scores were compared between LGG patients stratified by various clinicopathological features. The results demonstrated that LGG patients with the clinicopathological features of age ≥45 years, more malignant histological type (anaplastic oligodendroglioma/oligoastrocytoma), higher WHO grade, IDH wide type, 1p19q noncodel, and MGMT promoter unmethylated showed significantly higher levels of risk score, while no risk score differences were observed between patients satisfied by gender (Figure 5(a)). To determine whether the prediction power of the m^6^A-related prognostic signature retains in various subgroups, we performed subgroup survival analyses based on clinicopathological features (Figure 5(b)). The worse OS was noted in the high-risk groups regardless of age, gender, grade, and MGMT promoter status. Furthermore, patients with higher risk still had shorter OS than those with lower risk in the WHO grade III, anaplastic astrocytoma, anaplastic oligodendroglioma/oligoastrocytoma, IDH wide type, and 1p19q noncodel subgroups, except for the WHO grade II, astrocytoma, oligodendroglioma/oligoastrocytoma, IDH mutant, and 1p19q codel subgroups.

### 3.5. Establishment and Evaluation of a Nomogram Based on Independent Prognostic Indicators for OS

**Figure 6:**
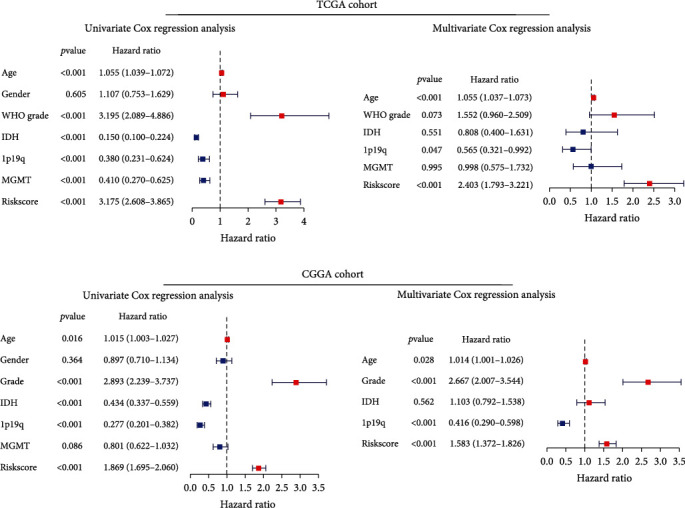
(a) Univariate and multivariate Cox regression analyses in the TCGA cohort. (b) Univariate and multivariate Cox regression analyses in the CGGA cohort.

**Figure 7:**
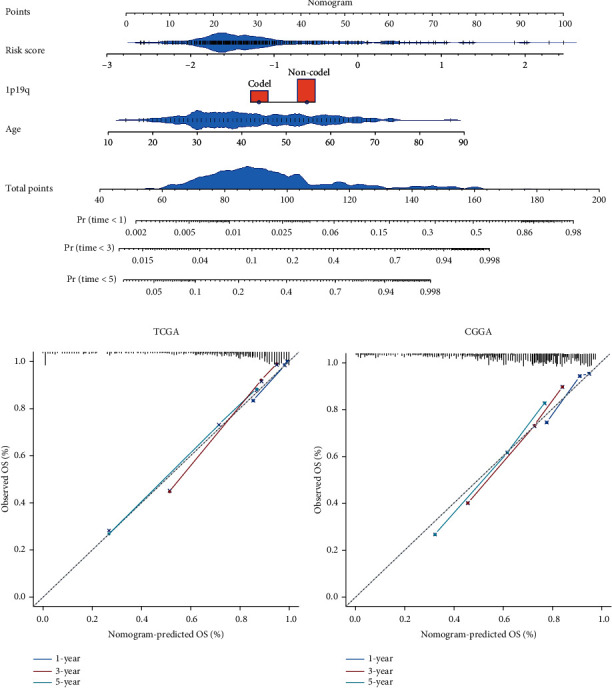
Establishment and evaluation of a nomogram. (a) A nomogram was established based on the signature-based risk score, age, and 1p19q codeletion status in the TCGA cohort. (b, c) Calibration plots of the nomogram for predicting the probability of 1-, 3-, and 5-year OS in the TCGA and CGGA cohorts.

To identify the independent prognostic indicators for OS, the OS-related factors identified by univariate Cox regression analyses were subsequently analyzed using multivariate Cox regression. The signature-based risk score was an independent prognostic indicator for OS in both TCGA and CGGA cohorts (both *p* < 0.001; Figures 6(a) and 6(b)). Then, a nomogram was established based on the independent prognostic indicators (age, 1p19q, and Risk score) in the TCGA cohort (Figure 7(a)). The C-indices of this nomogram were 0.73 ± 0.06 in the TCGA cohort and 0.75 ± 0.04 in the CGGA cohort. The calibration plots showed a perfect fit between the actual and nomogram-predicted probability of 1-, 3-, and 5-year OS in both cohorts (Figures 7(b) and 7(c)). Of importance, these results indicated that the nomogram had the potential to develop into a quantitative tool to predict the prognosis of LGG patients.

### 3.6. Correlation between the m^6^A-Related Prognostic Signature and the Immune Landscape of LGG Microenvironment

**Figure 8:**
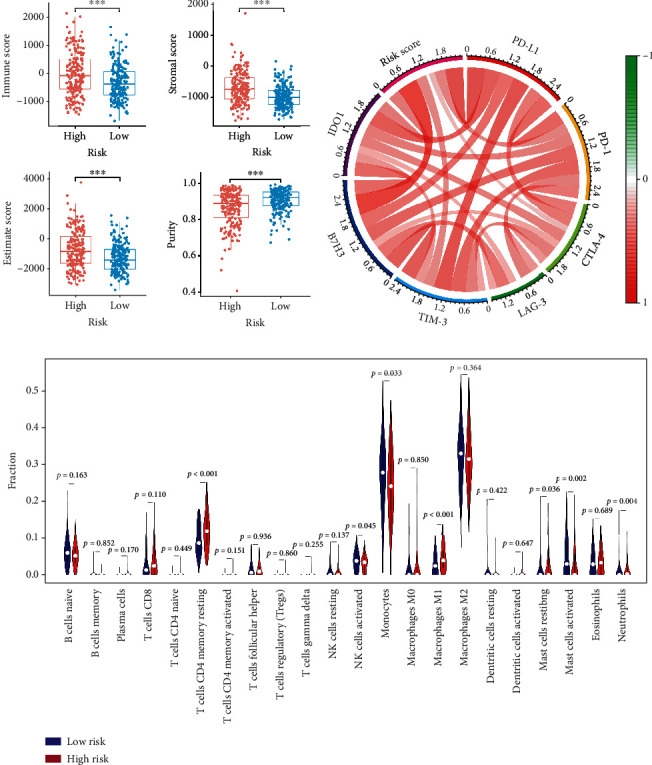
Correlation of the m^6^A-related prognostic signature with the immune landscape of LGG microenvironment in the TCGA cohort. (a) The comparison of immune scores, stromal scores, ESTIMATE scores, and tumor purity between the high- and low-risk groups. (b) The correlation between the risk score and the expression levels of immune checkpoints. (c) The abundance of 22 immune cells in the high- and low-risk groups. ^∗^*p* < 0.05, ^∗∗^*p* < 0.01, and ^∗∗∗^*p* < 0.001.

In the TCGA cohort, the high-risk group showed significantly higher immune, stroma, and ESTIMATE scores and lower tumor purity compared with the low-risk group (Figure 8(a)). Moreover, the risk score was correlated with the expression of immune checkpoints, including PD-1, PD-L1, CTLA-4, LAG-3, TIM-3, B7H3, and IDO1 (Figure 8(b)). Besides, different extents of immune cell infiltrations were observed in the high-risk group with lower abundance of activated NK cells, monocytes, resting mast cells, activated mast cells, and eosinophils but higher abundance of CD4+ memory resting T cells, M1-type macrophages, resting mast cells, and neutrophils (Figure 8(c)). These results confirmed a tight correlation between the m^6^A-related prognostic signature and the immune landscape of the LGG microenvironment.

## 4. Discussion

As more and more m^6^A RNA methylation regulators have been identified, the important roles played by m^6^A modification in cancers are being gradually unveiled. At present, the roles of individual genes in tumorigenesis of glioma via mediating m^6^A modification are the focus of intense research efforts. METTL3 regulates the proliferation, migration, and invasion of glioma cells by inhibiting PI3K/Akt signaling pathway [23]. ALKBH5 maintains tumorigenicity of GBM stem-like cells by sustaining FOXM1 expression and cell proliferation program [24]. Inhibiting the expression of FTO enhances the effect of temozolomide on glioma [25]. According to most relevant researches, no distinction was made for the different WHO grades of glioma, or GBM tended to receive more attention. Although LGG has a relatively low degree of malignancy compared with GBM, the high rate of postoperative recurrence and malignant progression should not be underestimated. Given the heterogeneity between LGG and GBM in tumor biological behavior, there is a need to comprehensively evaluate the prognostic role of m^6^A RNA methylation regulators in LGG.

In this study, a total of 36 m^6^A RNA methylation regulators were selected, most of which were found to be differentially expressed in LGG tissues compared with normal brain tissues and GBM tissues. There were large differences in the expression levels of m^6^A RNA methylation regulators even between WHO grade II and III. The differential expression profiles inspired us to further explore the role of m^6^A RNA methylation regulators in LGG. Afterwards, three clusters of LGG patients were identified through consensus cluster analysis based on the expression levels of 36 m^6^A RNA methylation regulators. The PCA and survival analysis confirmed the significant discrimination among the three clusters. Cluster 3 had worse survival outcomes and was closely related to malignancy-related biological processes and signaling pathways. Subsequently, an m^6^A-related prognostic signature was constructed. The Kaplan-Meier curves, ROC curves, and univariant and multivariant Cox regression analyses verified that this prognostic signature performed excellently in prognostic stratification of LGG patients. Furthermore, a nomogram for 1-, 3-, and 5-year OS were established based on the signature-based risk score combining age and 1p19q codeletion status. The C-indices and calibration plots suggested that this nomogram has the potential to be an effective assessment tool to identify personalized mortality risk for LGG patients. Finally, we uncovered the differential immune landscape between risk subgroups by comparing the immune, stromal, and ESTIMATE scores; tumor purity; expression levels of immune checkpoints; and abundance of immune cells.

The m^6^A-related prognostic signature contained nine m^6^A RNA methylation regulators, seven of which are “readers” (ZCCHC4, SETD2, IGF2BP2, IGF2BP3, YTHDF2, EIF3H, and YTHDC1) and the other two are “writers” (RBM15) and “erasers” (ALKBH3), respectively. Among these critical genes, several have been investigated to be associated with glioma. SETD2, a highly mutated gene, contributes to the tumorigenesis of high-grade glioma [26]. IGF2BP3 promotes glioma cell migration by enhancing the translation of RELA/p65 [27]. YTHDF2, phosphorylated and stabilized by EGFR/SRC/ERK, is required for cholesterol dysregulation, cell proliferation, invasion, and tumorigenesis of GBM [28]. However, there have been few reports focusing on LGG. Little information has been available regarding whether the m^6^A RNA methylation regulators contribute to the heterogeneity between LGG and GBM in tumor biological behavior, drug resistance, and prognosis. Therefore, we expect that our findings help to identify the prognostic m^6^A RNA methylation regulators in LGG and provide insights into their potential roles in LGG tumorigenesis and progression.

Tumor microenvironment (TME) has been identified as an essential regulatory role in the occurrence and progression of tumors [29]. It has been reported that LGG patients with high immune scores or high stromal scores had a poor prognosis [30–32]. Emerging evidence confirmed that the dysregulation of m^6^A RNA methylation regulators contributes to the heterogeneity of TME [12, 33, 34]. Up to now, the impact of m^6^A RNA methylation regulators on the immune landscape of the LGG microenvironment remains unclear. Firstly, this study found that LGG patients in the high-risk group had higher immune scores, higher stromal scores, higher ESTIMATE scores, and lower tumor purity than those in the low-risk group. Nowadays, immunotherapy, represented by immune checkpoint blockades (PD-1/L1), has been one of the most promising treatment strategies against various cancers. Interestingly, a positive correlation between the risk score and expression level of immune checkpoints was discovered. Thus, the risk stratification based on the m^6^A-related prognostic signature might help predict the efficacy of immune checkpoint blockades. As a critical part of the complex TME, immune cells have been identified to be associated with the tumor biological behavior and prognosis [35–37]. The results showed a lower abundance of activated NK cells, a major tumor killer cell type, in the high-risk group. Taken together, our findings suggested that m^6^A RNA methylation regulators partly participate in TME regulation of LGG and might provide new insights into the immunotherapy for LGG.

Undeniably, some limitations of our study should be pointed out. Firstly, the m^6^A-related prognostic signature was constructed and validated with retrospective data from public databases. Using prospective data to assess its clinical utility would be more convincing. Secondly, this study only focused on the transcriptome data, other data types like methylation, single nucleotide polymorphism (SNP), copy number variation (CNV), and protein level were not covered. In addition, due to the lack of experimental evidences, this study was not able to assess the role and molecular mechanism of individual regulators in depth. Further in vivo and in vitro experiments are essential to explore the regulatory mechanism of m^6^A RNA methylation regulators in LGG.

## 5. Conclusion

All in all, the present study systematically investigated the expression pattern, prognostic value, and effect on the immune landscape of m^6^A RNA methylation regulators in LGG. We identified three clusters that stratified the prognosis of LGG patients. An m^6^A-related risk signature was capable to precisely predict the prognosis of LGG patients and was correlated with the immune landscape of the LGG microenvironment. We hope that our findings provide comprehensive evidence for subsequent research about m^6^A modification in LGG.

## Figures and Tables

**Table 1:** Clinicopathological features of LGG patients in this study.

Characteristics		Training cohort	Validation cohort
		TCGA (*n* = 423)	CGGA (*n* = 590)
Age (years**)**	<45	245	398
	≥45	178	192
Gender	Female	189	250
	Male	234	340
WHO grade	II	201	269
	III	222	321
IDH status	Mutant	344	413
	Wild type	77	138
	NA	2	39
1p19q codeletion	Codel	141	180
	Noncodel	282	370
	NA	0	40
MGMT promoter status	Methylated	351	284
	Unmethylated	72	199
	NA	0	107

**Table 2:** The thirty-six m^6^A RNA methylation regulators.

	m^6^A RNA methylation regulators
Writes	METTL3, METTL14, WTAP, VIRMA, ZC3H13, CBLL1, RBM15, RBM15B, METTL16, PCIF1
Erasers	FTO, ALKBH5, ALKBH3
Readers	TRMT112, ZCCHC4, CPSF6, SETD2, HNRNPC, RBMX, HNRNPA2B1, IGF2BP1, IGF2BP2, IGF2BP3, YTHDC1, YTHDC2, YTHDF1, YTHDF2, YTHDF3, SRSF3, SRSF10, XRN1, FMR1, NXF1, PRRC2A, EIF3B, EIF3H

## Data Availability

Publicly available datasets were analyzed in this study. This data can be found here: The data analyzed in this study can be acquired in the TCGA (https://portal.gdc.cancer.gov/), CGGA (http://www.cgga.org.cn/) and GTEx (https://www.gtexportal.org) websites.

## Supplementary Materials

Supplementary Materials Supplementary Figure S1: consensus clustering analysis of 36 m^6^A RNA methylation regulators. (a, b) Relative change in area under CDF curve and consensus clustering CDF for *k* = 2 − 10. (c) Tracking plot for *k* = 2 to 9. (d) Consensus clustering matrix for *k* = 2.
