# Supplementary figures and images for: Identification of Critical m6A RNA Methylation Regulators with Prognostic Value in Lower-Grade Glioma
Source: Biomed Res Int. 2021 Jun 8;2021:9959212. doi: 10.1155/2021/9959212 (PMC8205593; doi:10.1155/2021/9959212)

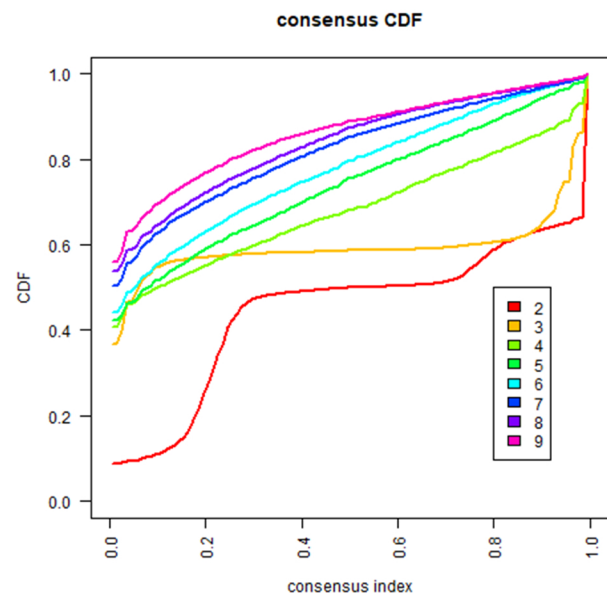

(a)

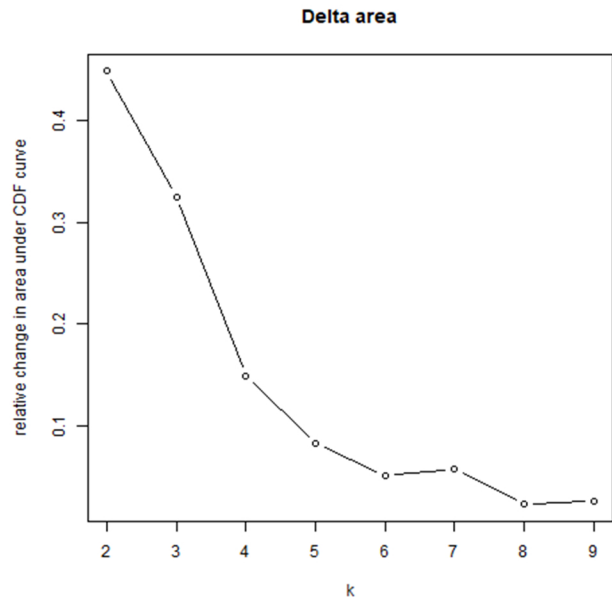

(b)

**tracking plot**

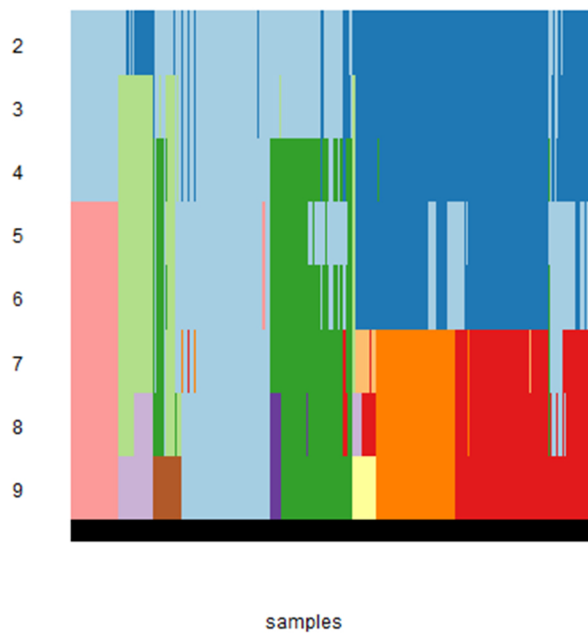

(c)

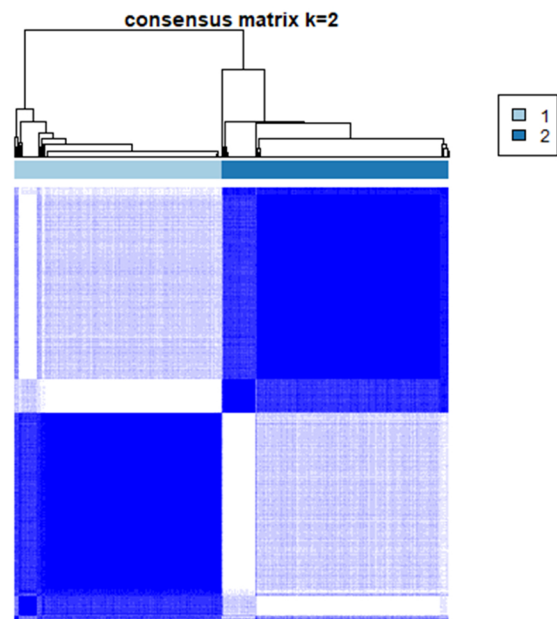

(d)

Supplement: Supplementary Materials — Supplementary Figure S1: consensus clustering analysis of 36 m6A RNA methylation regulators. (a, b) Relative change in area under CDF curve and consensus clustering CDF for k = 2 − 10. (c) Tracking plot for k = 2 to 9. (d) Consensus clustering matrix for k = 2. [file 9959212.f1.pdf]
